# Emotional Distraction and Bodily Reaction: Modulation of Autonomous Responses by Anodal tDCS to the Prefrontal Cortex

**DOI:** 10.3389/fncel.2015.00482

**Published:** 2015-12-18

**Authors:** Philipp A. Schroeder, Ann-Christine Ehlis, Larissa Wolkenstein, Andreas J. Fallgatter, Christian Plewnia

**Affiliations:** ^1^Department of Psychiatry and Psychotherapy, Neurophysiology & Interventional Neuropsychiatry, University of TübingenTübingen, Germany; ^2^Department of Psychology, University of TübingenTübingen, Germany; ^3^LEAD Graduate School, University of TübingenTübingen, Germany; ^4^Werner Reichardt Centre for Integrative NeuroscienceTübingen, Germany

**Keywords:** non-invasive brain stimulation, skin conductance response, cognitive control, autonomous nervous system, transcranial direct current stimulation, emotional distraction, automaticity

## Abstract

Prefrontal electric stimulation has been demonstrated to effectively modulate cognitive processing. Specifically, the amelioration of cognitive control (CC) over emotional distraction by transcranial direct current stimulation (tDCS) points toward targeted therapeutic applications in various psychiatric disorders. In addition to behavioral measures, autonomous nervous system (ANS) responses are fundamental bodily signatures of emotional information processing. However, interactions between the modulation of CC by tDCS and ANS responses have received limited attention. We here report on ANS data gathered in healthy subjects that performed an emotional CC task parallel to the modulation of left prefrontal cortical activity by 1 mA anodal or sham tDCS. Skin conductance responses (SCRs) to negative and neutral pictures of human scenes were reduced by anodal as compared to sham tDCS. Individual SCR amplitude variations were associated with the amount of distraction. Moreover, the stimulation-driven performance- and SCR-modulations were related in form of a quadratic, inverse-U function. Thus, our results indicate that non-invasive brain stimulation (i.e., anodal tDCS) can modulate autonomous responses synchronous to behavioral improvements, but the range of possible concurrent improvements from prefrontal stimulation is limited. Interactions between cognitive, affective, neurophysiological, and vegetative responses to emotional content can shape brain stimulation effectiveness and require theory-driven integration in potential treatment protocols.

## Introduction

Non-invasive brain stimulation has been shown to be effective in the modulation of cognitive control (CC) in healthy subjects and various psychiatric disorders (De Raedt et al., 2014; Kuo et al., 2014; Plewnia et al., 2015b). In particular, transcranial direct current stimulation (tDCS) has been demonstrated to reduce emotional biases in major depression under controlled conditions (Wolkenstein and Plewnia, 2013; Brunoni et al., 2014a). Therefore, by augmenting cortical activity in prefrontal regions, anodal tDCS can counteract disorder-specific CC deficits with possible positive effects on depression symptomatology (Fregni et al., 2006; Boggio et al., 2008; Ferrucci et al., 2009; Brunoni et al., 2013a; Segrave et al., 2014; Vanderhasselt et al., 2015). Central to intervention strategies that employ tDCS is the cognitive task performed during the stimulation, because task demands and cortical activation is associated with neuromodulatory effects (Andrews et al., 2011; Zwissler et al., 2014; Gill et al., 2015; Pope et al., 2015). In emotional tasks, goal-directed behavior can be disrupted by salient emotional information. In turn, specific activation of prefrontal regions can modulate and subordinate emotional responses to irrelevant distraction. In this context, PFC activity corresponds with the preservation of goal-directed cognitive performance (Anticevic et al., 2010; Wessa et al., 2013). At the same time, visceral feedback from autonomic responses to emotional distractors is critically integrated in cognitive processing (Critchley, 2005), and such overt or covert body-state structures are suggested to be relevant for human reasoning and decision making (Damasio et al., 1996). Accordingly, if a CC task requires the suppression of distracting emotional information, effective behavior is possibly deflected by arousing emotional information already on a vegetative level.

Explicit strategies to up- or downregulate negative emotional experience can modify responses of the autonomous nervous system (ANS; Eippert et al., 2007; Driscoll et al., 2009). Notably, emotion reappraisal efficacy can be augmented by anodal tDCS in both directions, and corresponding vegetative changes in skin conductance responses (SCRs) were documented (Feeser et al., 2014). However, in a natural context, emotional content often distracts effective processing automatically, engaging neural systems differentially: relative to reappraisal, distraction engaged less amygdala activity, but prefrontal, and parietal activation was increased (McRae et al., 2010). Consequently, the tight interplay of prefrontal and subcortical systems (Ochsner and Gross, 2005) as a basis of CC is also expressed in according responses of the ANS. In this context, SCRs were sensitive to cognitive conflicts, (aware) error commitment (O’Connell et al., 2007), and correlated with post-error behavioral adjustments (Hajcak et al., 2003; Kobayashi et al., 2007). To date, only few studies have reported effects of prefrontal tDCS on autonomic responses (Schestatsky et al., 2013). For instance, during blocks of emotional (negative and neutral) picture viewing, left anodal tDCS was associated with lower cortisol levels and higher high-frequency heart-rate variability (Brunoni et al., 2013b). In resting state, there was no autonomous response to tDCS with an extracephalic reference in either heart-rate (variability), respiratory rate, blood pressure, or sympatho-vagal balance (Vandermeeren et al., 2010). From different tasks, it was also noted that the effect of cathodal tDCS on impulsivity and truth-telling was associated with altered SCRs (Beeli et al., 2008; Karim et al., 2010). We reasoned that any modulation of ANS responses by prefrontal tDCS should be related to requirements of a task performed during the stimulation.

In contrast, there is good evidence on the efficacy of working memory improvements from anodal tDCS to the prefrontal cortex (Fregni et al., 2005; Andrews et al., 2011; see also: Kuo and Nitsche, 2012). Specifically, it has been demonstrated repeatedly that anodal tDCS improved reaction time and/or accuracy in the n-back task (Oliveira et al., 2013; Brunoni and Vanderhasselt, 2014; but see also: Steenbergen et al., 2015), a task that requires recurrent updating of working memory. To sum up, tDCS can modulate vegetative responses to emotional stimuli and the modulation of CC is most likely accompanied by vegetative reactions. With this study, we further focus on the interrelations of CC over emotional stimuli in a working memory task, in case of the modulation of SCRs to neutral and emotional distraction by prefrontal activity-enhancing, anodal tDCS.

More precisely, we closely follow up on the CC modulation over emotional content by prefrontal tDCS (Plewnia et al., 2015b). This intervention with anodal tDCS was described to ameliorate CC in depressive patients (Wolkenstein and Plewnia, 2013), while inhibitory, cathodal tDCS induced a depression-like attentional bias in healthy participants (Wolkenstein et al., 2014). An understanding of CC in emotional processing includes the goal-directed suppression of task-irrelevant processing, i.e., accurate working memory retrieval following the distraction by an emotional picture. Thus, not only prefrontal activation, but also its interplay with subcortical regions (i.e., the limbic system) and accompanied responses of the ANS must be considered. Crucially, whereas suppression of non-emotional content in other tasks (stop-signal task, conflict Stroop task) is also referred to CC processes, differential brain patterns might be involved. The modulation of such non-emotional CC processes by non-invasive brain stimulation might similarly rely on accompanied (medial-frontal) network activation (Yu et al., 2015). Here, and to better define the cognitive and physiological mechanisms involved in CC modulations over emotional distraction, it was suggested to also explore network loci of tDCS effects on affect regulation (Terhune and Cohen Kadosh, 2013). Thus, we report on recordings of electrodermal activity to emotional content during sham and anodal tDCS. More precisely, SCRs were selected as an appropriate measure compliant with a brief online emotional distraction paradigm, i.e., as opposed to more slow-paced ANS measures such as heart rate variability. During the stimulation, healthy participants solved a delayed-response working memory task. Negative, neutral, or positive content pictures of human scenes were presented in the delay period of the task to distract participants and thereby impair their cognitive-behavioral performance. A functional suppression of automatic emotional processing by CC was implicitly required by the task, and we hypothesized that SCRs should be attenuated by CC enhancing, anodal tDCS to the prefrontal cortex.

## Materials and Methods

The current study was part of a larger project evaluating the feasibility of CC modulations by tDCS in healthy and depressed individuals (Wolkenstein and Plewnia, 2013). Data on electrodermal activity and their modulation by tDCS are reported for the first time here and the data were gathered from a subgroup of healthy participants. We also re-analyzed the behavioral data for this subgroup in order to present a comprehensive picture of the experiment.

### Participants

A total of 22 healthy participants was recruited for the study, but electrodermal recordings failed in four cases due to technical problems. Thus, physiological data were available for 18 right-handed healthy participants (mean age = 31.3 years, *SD* = 2.5 years, three male). All participants were separately screened by a structured clinical interview for a history or presence of psychiatric disorder (SCID-I; Wittchen et al., 1997). Further exclusion criteria were assessed in the preceding diagnostic appointment and included: seizures, past or present neurologic conditions, metal objects in head-area, pacemaker, pregnancy, verbal IQ < 80, and insufficient knowledge of the German language. Healthy participants were invited to two experimental sessions (sham and anodal tDCS) in counterbalanced order on separate days. The experimental procedure was approved by the ethics committee of the University Hospital Tuebingen (approval ID: 211/2010BO1). All subjects gave written informed consent in accordance with the Declaration of Helsinki and received monetary compensation. Besides the general information given in the consent, all subjects were naïve with regard to the aim of the study.

### Experimental Design and Procedure

The study followed a cross-over, double-blinded, sham-controlled design. All participants underwent the two experimental conditions (sham, anodal tDCS) in counterbalanced order on separate days, but within 1 week. Screening for psychiatric conditions and exclusion criteria was carried out on a separate day before testing.

After preparation of stimulation and electrodermal recording, participants completed the Delayed-Response Working Memory task (Dolcos and McCarthy, 2006), a task that continuously requires CC over automatic emotional activation (McRae et al., 2010). In each trial of the task, six letters were presented as a row-sequence for 2 s. Participants were instructed to remember these letters throughout the trial. For the following 6 s, one interfering picture (negative, neutral, or positive content) was displayed. After the delay, a probe letter was presented for 4 s and participants had to indicate as fast as possible whether the probe letter was part of the memorized letter-sequence by pressing a keyboard button. The task consisted of 45 trials with equal contingencies for picture contents and was preceded by 10 additional exercise-trials. The exercise-trials were not included in the analyses. The same distractors were used in both sessions in randomized order. In order to reduce content-interference, all pictures displayed human scenes and centrally filled 2/3 of the screen. The pictures were taken from the standardized Emotional Picture Set (EmoPicS; Wessa et al., 2010). Participants rated all stimuli separately for pleasantness and arousal intensity on a 9-point Likert scale (1: negative/not at all arousing, 9: positive/very arousing).

### tDCS

Transcranial direct current stimulation was administered by a battery-driven CE-certified DC-Stimulator (NeuroConn GmbH, Illmenau, Germany) using a pair of saline-soaked 5 × 7 sponge electrodes (maximum current density: 0.02857 mA/cm^2^). The anode electrode was placed over the left dlPFC (F3 according to the international 10–20 system of electrode-placement; Jasper, 1958) and the cathode, return electrode was fixed to the right upper arm (M. deltoideus) to avoid an opposite polarization of another cortex area (Priori et al., 2008). Predefined codes assigned to either sham or verum stimulation were used to start the stimulation, effectively blinding both experimenter, and participant. For sham stimulation, the current was ramped down after 40 s (cf. Gandiga et al., 2006), which is known to elicit similar sensations to verum stimulation and to conceal possible motivational effects. Yet, blinding efficacy was not assessed explicitly. For verum stimulation (and the beginning of the sham stimulation), a constant current of 1 mA with a linear fade-in/fade-out phase of 5 s was administered for a duration of 20 min. The task started 5 min after stimulation onset, and thus participants completed the task parallel to verum stimulation, or started and completed the task after sham stimulation had ended. The order of sham and verum tDCS was counterbalanced across participants.

### Electrodermal Recordings

Electrodermal activity was continuously recorded (sampling rate: 5000 Hz) by two Ag/AgCI skin electrodes of a BrainVision GSR MR module (Brain Products GmbH, Munich, Germany) with a constant voltage of 0.5 V. Bilateral electro-dermal activity was derived from the index- and ring finger of the left hand with isotonic paste (Mansfield R&D, St. Albans, VT, USA). The measured curve was segmented oﬄine for the emotional conditions of the task. SCRs for clear peaks were determined within a range of 6 s after stimulus onset. Manual corrections were conducted for unspecific reactions and artifacts were rejected (unnatural large amplitudes (>2 μs), reactions before stimulus presentation or steep, sudden rise or drop of the curve). The mean difference between peak and plateau (baseline-corrected, 500 ms pre stimulus) of all artifact-free episodes for each condition and participant was determined as SCR amplitude and submitted to further analyses. SCR amplitudes were log-transformed to normalize their distribution.

### Data Treatment

Mean valence and arousal ratings for positive, neutral, and negative pictures were pairwise compared. We also analyzed accuracy rates in the delayed-response working memory task using paired *t*-tests for the effect of tDCS on performance distractions by each of the three valence levels. This analysis was chosen to confirm the previously reported findings from a larger sample (Wolkenstein and Plewnia, 2013).

For electrodermal recordings, the individual SCR amplitude was extracted and submitted to a 2 × 3 repeated measures analysis of variance (ANOVA) comprising the factors *stimulation*_VERUM,SHAM_ and *valence*_pos,neg,neu_. We followed up on significant findings with paired *t*-tests for each level of valence. Effect sizes and 95% confidence intervals are reported for follow-up paired *t*-tests (Lakens, 2013). The alpha level was set at α = 0.05.

To investigate associations between individual performance variations in the delayed-response working memory task and SCR responses, we computed the within-subjects correlation across all conditions (Bland and Altman, 1995). To further specify the association between performance and SCR modulations by tDCS, we also performed an exploratory hierarchical multiple regression analysis on the change scores. In this additional analysis, we were interested in the interrelations of stimulation effects: are SCR modulations necessarily followed by performance improvements, or is the range of effective tDCS modulations limited? To characterize this relationship, we performed a hierarchical model selection: change in performance was the dependent variable, and mean-centered change in SCR on a linear (Step 1) and quadratic term (Step 2) were predictor variables. In Step 3, the categorical variable *valence*_POS,NEG,NEU_ was transformed to two dummy-coded indicator variables and these predictors were added to a full model. Following theory-motivated model selection, we tested the coefficients for the included predictors. Tests for normal distributions on the continuous measures signaled no deviations (*p*s > 0.31). The Breusch–Pagan test for heteroscedacity was not significant (Chi^2^ = 8.02, *p* = 0.09).

## Results

### Behavioral Data

Self-report ratings for positive, neutral, and negative picture valence and arousal are shown in **Table 1**. For valence, the ratings followed the expected pattern with highest scores of pleasantness for positive and lowest scores for negative pictures. For arousal, neutral pictures were reported as less arousing than negative and positive pictures, and negative pictures aroused participants the most. All pairwise comparisons yielded significant differences in valence ratings, *p*s < 0.001, and in arousal ratings, *p*s < 0.002.

**Table 1:** Ratings for Valence and Arousal were given on a 9-point Likert scale (1: negative/not at all arousing, 9: positive/very arousing).

	Negative *M* (*SE*)	Neutral *M* (*SE*)	Positive *M* (*SE*)
Valence	2.23 (0.18)	4.76 (0.23)	7.04 (0.22)
Arousal	5.90 (0.32)	1.74 (0.22)	4.10 (0.30)

**FIGURE 1:**
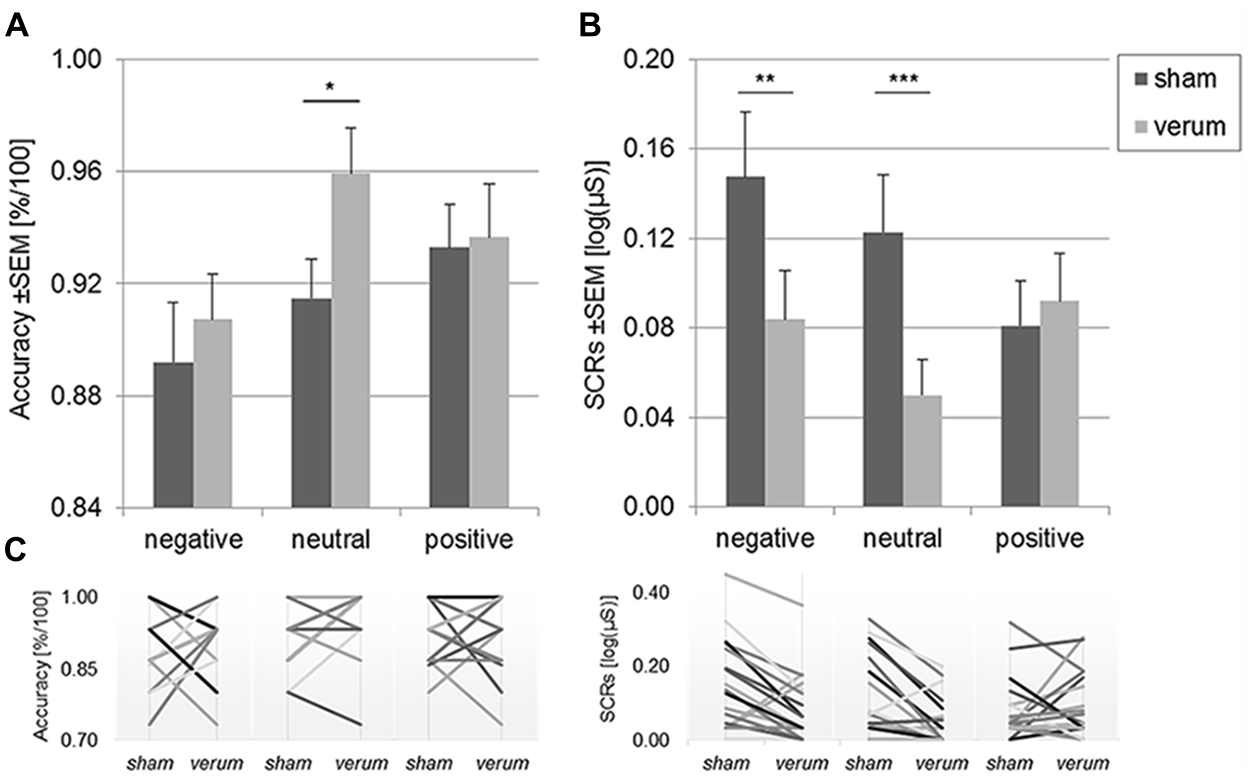
**Mean accuracy rates (A) and skin conductance responses (SCRs; B) following negative, neutral, and positive content during sham and anodal tDCS.** Panel **(C)** displays individual changes for each stimulation condition and content for each subject. Accurate responding was enhanced by anodal tDCS following neutral content only. SCRs to negative and neutral content were diminished by the stimulation. Error bars reflect standard errors of the mean (SEM). Note that **(C)** dismisses overlapping responses. ^∗^*p* < 0.05, ^∗∗^*p* < 0.01, ^∗∗∗^*p* < 0.005.

Mean accuracy rates for the three valence levels and two stimulation conditions are depicted in **Figure 1A**. Participants gave more accurate responses during anodal stimulation following neutral pictures, *t*(17) = 2.61, *p* = 0.018, *d* = 0.64 [95% CI: 0.006; 0.078]. For emotional (positive and negative) stimuli, there was no effect of tDCS, *p*s > 0.59. Thus, the analysis for this subgroup corresponds with the general behavioral findings for healthy participants reported before (Wolkenstein and Plewnia, 2013).

### Electrodermal Activity

Mean SCRs to negative, positive, and neutral content as a function of the stimulation condition (sham vs. anodal tDCS) are displayed in **Figure 1B**. The data were submitted to a 2 × 3 repeated measures ANOVA comprising the factors *valence*_POS,NEG,NEU_ and *stimulation*_VERUM,SHAM_. There was a significant main effect of *stimulation*_VERUM,SHAM_, *F*(1,17) = 8.70, *p* = 0.009, $\eta_{p}^{2}$ = 0.34, but the main effect of *valence*_POS,NEG,NEU_ was not significant, *F(2,16) = 2.03, p* = 0.15. However, a two-way interaction of *stimulation*_VERUM,SHAM_ and *valence*_POS,NEG,NEU_ emerged, *F(2,16) = 5.22, p* = 0.011, $\eta_{p}^{2}$ = 0.24. We followed up on this result with paired *t*-tests for each of the three valence types.

Anodal tDCS significantly reduced SCR amplitudes following negative content, *t*(17) = 2.94, *p* = 0.009, *d* = 0.56 [95% CI: 0.018; 0.109], and following neutral content, *t*(17) = 3.67, *p* = 0.002, *d* = 0.74 [95% CI: 0.031; 0.115]. Notably, there was no modulation of responses to positive content, *t*(17) = 0.46, *p* = 0.65. As a consequence, SCRs to positive pictures were marginally larger than SCRs to neutral pictures during anodal tDCS, *t*(17) = 2.14, *p* = 0.048, *d* = 0.51 [95% CI: -0.0002; 0.0838], but they did not differ in amplitude to SCRs to negative pictures, *t*(17) = 0.37, *p* = 0.72. In contrast, during sham tDCS, the difference between negative and positive picture SCRs was significant, *t*(17) = 3.10, *p* = 0.006, *d* = 0.59 [95% CI: 0.0212; 0.1114]. The remaining paired differences in the sham and verum sessions were not significant, *p*s > 0.08.

### Within-Subjects Correlation: Functional Relevance of SCR for Performance

The Bland-Altman within-subjects regression coefficient associated with SCR and performance was significant, *b* = -0.161, *p* = 0.027 [95% CI: -0.304; -0.018], and a negative coefficient signaled individual performance increases with SCR decreases, *r* = -0.214.

### Exploratory Analysis: Functional Relevance of SCR Modulation

**FIGURE 2:**
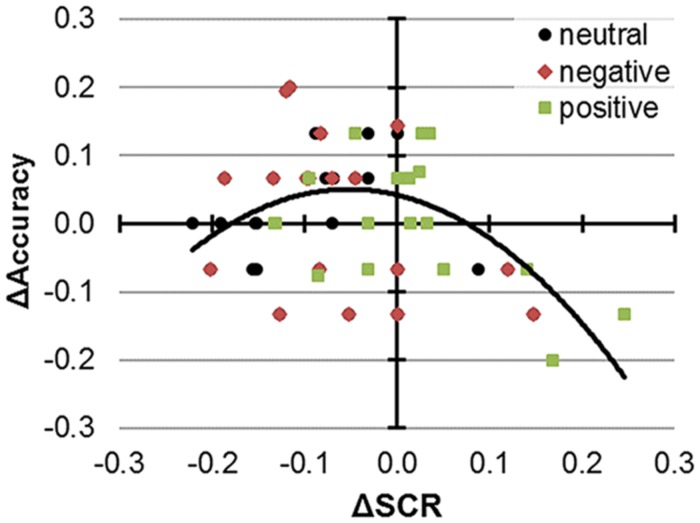
**Performance and SCR modulations by 1 mA anodal tDCS.** Color and shape indicate responses to neutral, negative, and positive stimuli. In the corresponding regression model, the depicted quadratic term and the intercept were significant predictors of performance changes and there was no additional variance explained by valence.

Finally, we asked whether SCR and performance *modulations* by excitatory stimulation were interconnected. We first hypothesized that performance improvements and SCR attenuations by anodal tDCS as outlined above could directly relate. However, a simple regression model (within-subjects) with Δ*SCR*_LINEAR_ as mean-centered predictor for Δ*Accuracy* was not significant, *F*(2,53) = 1.77, *p* = 0.18, *R*^2^_adjusted_ = 0.03. We next hypothesized the range of effective tDCS modulations on both SCR and performance to be limited, i.e., because – artificially stimulated – large as opposed to small SCR modulations might become detrimental to CC performance (i.e., Kobayashi et al., 2007). Thus, we next included a quadratic term Δ*SCR*_SQUARED_ (**Figure 2**). The corresponding model fit improved tremendously, Δ*F* = 12.66, *p* = 0.001, Δ*R*^2^_adjusted_ = 0.19. Further inclusion of valence in a full model did not yield significant improvements, Δ*F* = 0.498, *p* = 0.611, Δ*R*^2^_adjusted_ = 0.02. Whereas we already outlined direct effects and interactions of valence on performance and SCR, the relationship between SCR and performance please emphasize seemed akin across the valence conditions.

After model selection, we proceeded with testing the predictors. With regard to the best regression model including both linear and quadratic predictors, *F*(3,53) = 5.67, *p* = 0.002, *R*^2^_adjusted_ = 0.209, negative-signed coefficients emerged for both predictors, and Δ*SCR*_SQUARED_ was significant, *b* = -3.158, β = -0.482, *p* = 0.001 [95% CI: -4.941; -1.376], whereas Δ*SCR*_LINEAR_ was not significant, *b* = -0.063, β = -0.065, *p* = 0.63. Also, the intercept was significantly shifted, *b* = 0.057, *p* = 0.042 [95% CI: 0.002; 0.112]. Given that the predictors were mean-centered, the significant intercept estimates approximate performance improvements from anodal tDCS by 5.7 ± 2.7% in cases were the vegetative response was reduced by a mean ΔSCR of 1.128 ± 0.240 μs. According to the model, both additional in- or decreases in SCR diminished the performance, outlining an inverse-U shape functional relationship.

## Discussion

Our results demonstrate that non-invasive brain stimulation, i.e., anodal tDCS to the prefrontal cortex, can modulate automatic vegetative responses (SCR amplitudes) of healthy participants to negative and neutral distractors in a working memory task. In this study, the already smaller SCR amplitudes to positive distractors were not modulated by tDCS. Notably, individual working memory performance, indicative of the amount of distraction by emotional information, was associated with SCR amplitude.

For the trials with emotional distractors, no behavioral improvements by tDCS were detected (cf. Wolkenstein and Plewnia, 2013), but SCRs to negative distractors were reduced to a level of positive distractors. Consequently, our exploratory regression analysis revealed an inverse U-shape relationship between performance and SCR modulations by tDCS, suggesting that a reduction of distraction in the working memory task was associated with a specific SCR reduction. Further cross-validation of this model with different stimuli and additional physiological measures (i.e., heart-rate variability, oscillatory brain activity) will be necessary to clarify the generality of this association and to refine the cognitive-physiological underpinnings of CC modulations. Also, whereas our results are suggestive of an effect of anodal tDCS, further active control conditions are needed to account for general vs. polarity-specific stimulation effects. Nevertheless, our findings provide preliminary evidence for a functional relevance of ANS modulations for the effects of tDCS on CC. Moreover, SCR modulations by tDCS may index subtle effectiveness of this intervention. However, given that tDCS effects on ANS have been scarcely investigated before (Schestatsky et al., 2013), further studies are needed to investigate outstanding ambiguities and to also establish the integration of other indices such as heart-rate variability into corresponding paradigms.

For effective CC over emotional deflection, several distinct mechanisms were described (Ochsner and Gross, 2005). First, voluntary regulation of emotional intensity can activate reappraisal strategies. The effectiveness of anodal tDCS to the right prefrontal cortex in voluntary emotion regulation was underscored by corresponding changes in vegetative responses (Feeser et al., 2014). Second, the automatic activation of an emotional response also involves vegetative responses (Bradley et al., 1993), and our study provides first evidence that this mechanism is malleable by prefrontal tDCS.

Considering the accumulating evidence that CC processes for emotional and non-emotional conflicts dissociate (Soutschek et al., 2013), it is likely that suppression of distractive neutral content recruits different pathways than suppression of distractive emotional content. Our findings support this notion by providing both behaviorally and physiologically distinct effects of the stimulation. Notably, our results differ from findings reported in MDD patients that typically show improvements in emotional processing following excitatory stimulation (Leyman et al., 2011; Brunoni et al., 2014a,b; Plewnia et al., 2015b). In addition, both behavioral and autonomous effects of non-invasive brain stimulation, i.e., cortisol concentration, can largely dissociate and even reverse in (sub-)clinical or trait-specific differing populations (Sarkar et al., 2014). In this line, while performance in a non-emotional working memory task was enhanced by tDCS in both healthy and depressed individuals, MDD patients exclusively presented improvements in emotional cognition from prefrontal anodal stimulation (Moreno et al., 2015). Similarly, in our study, the direct behavioral effect of tDCS was restricted to neutral pictures in healthy participants. In SCRs, however, we are able to document more subtle changes in vegetative responses.

One shortcoming of our results is that SCRs to positive pictures were already smaller than to negative pictures during the sham-session and thereof, likely, we did not observe a modulation of SCRs to positive content by tDCS. At first, this pattern seems inconsistent with the established view that arousal, but not valence drives autonomous responses (Bradley et al., 2001; Lang, 2014). However, valence-driven ANS responses produced rather ambiguous results in the literature (Kreibig, 2010): for instance, regarding positive affect, SCRs varied largely depending on the emotional subtype, such as joy, happiness, or contentment. Similar to our results, non-human, potential phobic, and neutral stimuli produced equally large SCRs in a previous study (Maltzman and Boyd, 1984) and similar SCR responses to negative and neutral, but not positive facial expressions were described (Vrana and Gross, 2004; but see: Alpers et al., 2011). Integrating our other observations, both cortical and vegetative responses to emotional content that eventually determine tDCS effectiveness likely depend on rather specific task and individual characteristics, i.e., genotype (Nieratschker et al., 2015). Also, the individual interpretation of emotional distraction is variable and no specific regulation instruction was given in our study, limiting the conclusions we can draw from this unexpected pattern of results.

The prefrontal cortex is involved in a multitude of functions, and tDCS applications proved effective in modulating various aspects of cognition besides affect regulation (Kuo and Nitsche, 2015). Attributing the tDCS effect reported in this study to a general modification of working memory, a widely accepted mechanism (Brunoni and Vanderhasselt, 2014), however, comes short of the exact requirements of the task, that is, updating and retrieval of a letter string series was possibly perturbed by the intermediate presentation of emotional pictures. It follows that in this specific working memory task it is necessary to down-regulate emotional (including autonomous) responses to task-irrelevant content in order to correctly identify the probe letter. Thus, our results correspond to both effects of tDCS on emotion reappraisal (Feeser et al., 2014) and CC in a larger sense (Beeli et al., 2008; Karim et al., 2010).

Previous studies on tDCS effectiveness have focused more on cortical than on limbic and vegetative processes. However, considering that cognitive processes constantly integrate visceral feedback, studies on vegetative consequences of a stimulation may offer further insights into the mechanisms of CC involved in emotional (dis-)engagement (Sequeira et al., 2009). While emotional states are reflected in various autonomous indices, their causal directionality is not self-explanatory and afferent information can also influence bodily emotional state (Critchley, 2005). Excitatory stimulation to prefrontal regions might facilitate an early retraction from the visceral-cognitive feedback loops following task-induced emotions, and synchronous performance-emotion improvements by anodal tDCS have been documented (Plewnia et al., 2015a). In the presented setting and in related training paradigms, both network effects of affect regulation and visceral integration may interact with potential modulations by non-invasive brain stimulation.

## Conclusion

We outlined that vegetative responses to emotional and neutral stimuli can be modulated by non-invasive brain stimulation. In the CC paradigm applied, SCRs to negative and neutral distractors were significantly diminished by anodal as compared to sham tDCS. A possible functional relevance of somatic responses for efficient control of emotional distraction was outlined, and the preliminary evidence for modulatory associations suggests an inverse-U relation between performance and ANS modulations by tDCS. The study thereby augments our knowledge on the interrelations between cognitive-affective and physiological processes and their malleability by prefrontal stimulation. Future studies may investigate the physiological and neurophysiological underpinnings of CC enhancement by non-invasive brain stimulation, their polarity-specificity and exploit cognitive–visceral interrelations in clinical applications.

## Author Contributions

CP and LW designed study, CP and LW collected data, PS, LW, AE, and CP analyzed data, PS, AF, LW, AE, and CP contributed to the manuscript.

## Conflict of Interest Statement

The authors declare that the research was conducted in the absence of any commercial or financial relationships that could be construed as a potential conflict of interest.
